# mRNA expression of somatostatin receptor subtypes *SSTR-2*, *SSTR-3*, and *SSTR-5* and its significance in pancreatic cancer

**DOI:** 10.1186/s12957-015-0467-z

**Published:** 2015-02-13

**Authors:** Muhammad Shahbaz, Fang Ruliang, Zhang Xu, Liang Benjia, Wang Cong, He Zhaobin, Niu Jun

**Affiliations:** Department of Hepatobiliary Surgery, Qilu Hospital, Shandong University, Wenhua west Road #44, Jinan, 250012 China; Key Laboratory of Cardiovascular Remodeling and Function Research, Chinese Ministry of Education and Public Health, Jinan, 250012 Shandong People’s Republic of China; Department of Radiation Oncology, Qilu Hospital, Shandong University, Jinan, 250012 China

**Keywords:** Pancreatic cancer, Somatostatin receptor, mRNA

## Abstract

**Background:**

The aim of this study is to investigate the expressions of somatostatin receptor (*SSTR*), *SSTR-2*, *SSTR-3*, and *SSTR-5*, in pancreatic tissue and non-cancerous tissue and elucidate their clinical significance.

**Methods:**

The expression of somatostatin receptor subtypes *SSTR-2*, *SSTR-3*, and *SSTR-5* messenger RNA (mRNA) in 108 cases of cancer tissue and adjacent tissue in patients with pancreatic cancer was detected by reverse transcriptase polymerase chain reaction (*RT-PCR*). Expression of *SSTR-2*, *SSTR-3*, and *SSTR-5* mRNA was evaluated after specimens were taken from selected patients who underwent surgical resection by Whipple’s operation. We speculated the clinical significance of the expression of somatostatin receptor (*SSTR*) subtype genes *SSTR-2*, *SSTR-3*, and *SSTR-5* in pancreatic tissue and non-cancerous tissue and further elucidated their clinical significance.

**Results:**

The expression rates of *SSTR-2* mRNA in cancer and adjacent tissue of 108 patients with pancreatic cancer were 81.5% (88/108) and 97.2% (105/108), respectively; *SSTR-3* mRNA expression rates were 69.4% (75/108) and 55.6% (60/108). *SSTR-5* mRNA expression rates were 13.0% (14/108) and 18.5% (20/108).

**Conclusion:**

We propose that *SSTR-2* plays an important role in clinical implications for patients with pancreatic cancer undergoing somatostatin or its analog therapy.

**Electronic supplementary material:**

The online version of this article (doi:10.1186/s12957-015-0467-z) contains supplementary material, which is available to authorized users.

## Background

Somatostatin is a type of peptide hormone which has a wide range of biological functions. Recent studies have indicated that somatostatin and its analogs have obvious anti-proliferative effects on various solid tumors, such as gastroenteropancreatic neuroendocrine tumors and breast cancer, which are mediated through somatostatin receptors [1,2], but some clinical research have indicated that the impact of somatostatin and its analog receptor expression levels on outcomes in patients with pancreatic neuroendocrine tumors (*PNETs*) has not been evaluated [3,4]. Thus, whether or not there is expression of somatostatin receptor (somatostatin receptor, *SSTR*) in tumor tissue is an important underlying factor affecting the efficacy of somatostatin therapy. In this study, we detected the expression of somatostatin receptor subtypes *SSTR-2*, *SSTR-3*, and *SSTR-5* messenger RNA (mRNA) from 108 cases of cancer tissue and adjacent tissue in patients with pancreatic cancer using RT-PCR method. Thereafter, we explored the clinical implications of somatostatin therapy for patients with pancreatic cancer. Our study is aimed at exploring the cause from different therapeutic effects of somatostatin and its analogs on human pancreatic cancer and their relationship between changes of *SSTR-2* gene expression and expression of *SSTR-2*, *SSTR-3*, and *SSTR-5* genes [5].

## Methods

### Patient population and data collection

Our study was a retrospective analysis of patients who had been diagnosed pathologically as having pancreatic ductal adenocarcinoma by the Department of Pathology in Qilu hospital, between January 2010 and June 2013. The demographic, clinical, laboratory, and radiological data for all patients admitted or transferred to our hospital with a diagnosis of pancreatic ductal adenocarcinoma were reviewed retrospectively for this study. Included in this study, patients with pancreatic cancer must have received surgical resection as the initial treatment modality without major perioperative complications, have adequate archived tissue kept, and have complete clinicopathologic data obtained. This resulted in a collection of tissue from 108 patients, 72 males and 36 females with a median age of 55.7 years and an age range of 33 to 71 years. Among those, 85 had cases of carcinoma of the head of the pancreas and 23 cases of carcinoma of the body and tail of the pancreas. For *UICC TNM* clinical stages: stages I to II had 33 cases, and stages III to IV had 75 cases. We also collected the non-cancerous pancreatic tissue from 10 patients (all by excision of the normal pancreas were taken from surgical specimens) in order to compare the mRNA expression rate with the cancerous pancreatic tissues and adjacent normal tissue. The pathologic tumor-node-metastasis (TNM) classification was based on the criteria of the International Union Against Cancer (2009). The study protocol was approved by the Ethics Committee of Qilu Hospital affiliated to Shandong University. Informed consent was obtained from each patient prior to study enrollment at the time of hospital admission, and the detailed case characteristics are summarized in Table 1.

**Table 1:** **The basic characteristics of patients in this study**

**Clinical pathological factors**	***n*** ** 108**	**SSTR-2 mRNA**		***P*** **value**	**SSTR-3 mRNA**		***P*** **value**	**SSTR-5 mRNA**		***P*** **value**
		**Positive (** ***n*** **= 88)**	**Negative (** ***n*** **= 20)**		**Positive (** ***n*** **= 75)**	**Negative (** ***n*** **= 33)**		**Positive (** ***n*** **= 14)**	**Negative (** ***n*** **= 94)**	
Gender				*P* < 0.05^**^			*P* < 0.05^**^			*P* > 0.05
Male	56	40	16		33	23		8	48	
Female	52	48	4		42	10		6	46	
Age (years)				*P* > 0.05			*P* > 0.05			*P* > 0.05
<60	40 68 36 32 22 46	35 68 36 32 22 46	5		27 68 36 32 22 46	13		5	35	
≥60	68	53	15		48	20		9	59	
T stage				*P* > 0.05			*P* > 0.05			*P* > 0.05
T1	1	0	1		0	1		0	1	
T2	13	11	2		9	5		2	11	
T3	45	36	9		35	10		5	40	
T4	49	41	8		32	17		7	42	
N stage				*P* > 0.05			*P* > 0.05			*P* > 0.05
N0	63	53	10		42	21		7	56	
N1	34	28	6		25	9		5	29	
N2	11	7	4		8	3		2	9	
M stage				*P* > 0.05			*P* > 0.05			*P* > 0.05
M0	84	67	17		58	26		9	75	
M1	24	21	3		17	7		5	19	
TNM stage				*P* > 0.05			*P* > 0.05			*P* > 0.05
I to II	40	31	9		27	13		6	34	
III to IV	68	57	11		48	20		8	60	
Differentiation				*P* > 0.05			*P* > 0.05			*P* > 0.05
Well	40	33	7		30	10		4	36	
Moderate	45	35	10		29	16		5	40	
Poor/undifferentiated	23	20	3		16	7		5	18	
Survival (60-month follow-up)				*P* < 0.05^*^			*P* > 0.05			*P* > 0.05
Death	90	70	20		63	27		11	79	
Censored	18	18	0		12	6		3	15	

The expression of SSTR-2 was significantly relevant with the survival rate. The survival rate of patients with positive expression was higher than those with negative expression (*P* < 0.05^*^). The expressions of SSTR-2 mRNA and SSTR-3 mRNA were relevant with gender. Female positive rate was significantly higher than male (*P* < 0.05^**^). These three kinds of mRNA expression had no obvious relationships with age, TNM stage, and differentiation (*P* > 0.05).

### Reagents

Total RNA extraction reagents were bought from Promega (Madison, WI, USA). Specific primers, RT-PCR reagents and restriction enzymes, were provided by TaKaRa (Bio Company, Kyoto, Japan).

### Specimen collection

Non-necrotic cancer tissue and adjacent normal tissue (3 cm from cancer tissue) were taken from surgical specimens (non-cancerous pancreatic tissues were taken from the normal tissue). All were frozen in liquid nitrogen immediately after removal and placed in −80°C.

### RT-PCR

Complementary DNA (cDNA) was synthesized by reverse transcription of 20 μl total RNA in a reaction system. Following reaction system was used: MgCl_2_ 5 mmol/L, dNTPs 5 mmol/L, RNAsin 20U, AMV reverse transcriptase XL2.5U, oligo d (T) adaptor primers 2.5 μmol/L. The reaction was carried out at 65°C for 10 min; after cooling at 4°C for 5 min, AMV reverse transcriptase Xl was added and then incubated at 42°C for 60 min, 99°C heating for 5 min to inactivate the reverse transcriptase. PCR was performed using the following schedule: 30 cycles of denaturation at 94°C for 30 s; annealing of *SSTR-2* and *SSTR-5* at 57°C for 30 s and *SSTR-3* at 63°C for 60 s; positive control at 60°C for 30 s; extension at 72°C for 1 min; frozen at 4°C for 5 min. Special primer pairs were listed as supplementary Table 2.

**Table 2:** **Special primer pairs for amplifying human SSTR-2, SSTR-3, and SSTR-5 subtypes by RT-PCR**

**Primers**	**Sequences of primers**	**Size of PCR products (bp)**
SSTR-2		
Sense	5′-CATCATTGGGTTGTGTGCA-3′ (162 to 181)	256
Antisense	5′-GCTCGATGCTCATGACTGTC-3′ (399 to 418)	
SSTR-3		
Sense	5′-TGGCATTGGGTTGTTCATG-3′ (263 to 282)	435
Antisense	5′-CTTCACCAGGGTTGTGTGTAG-3′ (675 to 696)	
SSTR-5		
Sense	5′-GTTCATTGGGTTGTGTGCG-3′ (169 to 187)	101
Antisense	5′-CAACATTGGGTTGTGTGCC-3′ (262 to 381)	
Positive control		
F-1	5′-CATCATTGGGTTGTGTGCA-3′	462
R-1	5′-CATCATTGGGTTGTGTGCA-3′	

### Statistical method

All statistical data was evaluated by χ2 test. The *P* < 0.05 was considered statistically significant while three samples were compared. While comparing two samples, we used partitions of χ2 method to significant level α’. *α*’ = *α*/((k − 1) × *k*/2 + 1) (*k*, numbers of combination of any two). We could calculate the outcome of α’ is 0.0125. Thus, *P* < 0.0125 was considered statistically significant. Survival analyses were conducted by the life table method and the log-rank test. *P* values <0.05 were considered to be statistically significant. Survival curves were generated through the life table method.

## Results

In 108 cases of pancreatic cancer patients, the expression rate of *SSTR-2* receptor mRNA in adjacent cancer tissues was 97.2% (105/108) and in cancer tissue was 81.5% (88/108) (Table 3). The rate of positive *SSTR-2* mRNA expression among carcinous tissue, adjacent tissue of cancer, and non-carcinous pancreatic tissues were significantly different via analysis (*P* < 0.01). When compared between any two groups, the difference between carcinous tissue and adjacent tissue was also significant. Positive expression in carcinous tissue was also higher than adjacent tissue (*P* < 0.0125) (Table 3). Analysis from other groups did not show obvious differences (*P* > 0.0125). The expression rate of *SSTR-3* receptor mRNA in adjacent cancer tissue was 55.6% (60/108). In cancer tissue, it was 69.4% (75/108) (Table 3). The expression rate of *SSTR-5* receptor mRNA in adjacent cancer tissues was 18.5% (20/108). In cancer tissues, it was 13% (14/108) (Table 4). *SSTR-2*, *SSTR-3*, and *SSTR-5* mRNA expression rate of ten cases of non-cancerous pancreatic tissue were 100.0% (10/10), 70.0% (7/10), and 10.0% (1/10) (Table 2).

**Table 3:** **SSTR-2, SSTR-3, and SSTR-5 mRNA expression in different tissues**

**Tissue**	**Positive (** ***n*** **)**	**Negative (** ***n*** **)**	**Total**	**Positive rate**
*SSTR-2 mRNA expression* Carcinous tissues	88	20	108	81.5%
Adjacent cancerous tissues	105	3	108	97.2%
Non-carcinous pancreatic tissues	10	0	10	100%
Total	203	23	226	89.8%
*SSTR-3 mRNA expression* Carcinous tissues	75	33	108	69.4%
Adjacent cancerous tissues	60	48	108	55.6%
Non-carcinous pancreatic tissues	7	3	10	70%
Total	142	84	226	62.8%
*SSTR-5 mRNA xpression* Carcinous tissues	14	94	108	13.0%
Adjacent cancerous tissues	20	88	108	18.5%
Non-carcinous pancreatic tissues	1	9	10	10%
Total	35	191	226	15.5%

**Table 4:** **The χ** ^**2**^ **test outcomes of SSTR-2, SSTR-3, and SSTR-5 mRNA expression**

**Samples**	**χ** ^**2**^	***P***
*SSTR-2 mRNA expression* Three groups	15.82	*P* < 0.01
Carcinous tissues and adjacent cancerous tissues cancer	14.06	*P* < 0.0125
Carcinous tissues and non-carcinous tissues	2.23	*P* > 0.0125
Adjacent cancerous tissues and non-carcinous tissues	0.29	*P* > 0.0125
*SSTR-3 mRNA expression* Three groups	4.69	*P* > 0.05
Carcinous tissues and adjacent cancerous tissues	4.44	*P* > 0.0125
Carcinous tissues and non-carcinous tissues	0.0013	*P* > 0.0125
Adjacent cancerous tissues and non-carcinous tissues	0.78	*P* > 0.0125
*SSTR-5 mRNA expression* Three groups	1.51	*P* > 0.05
Carcinous tissues and adjacent cancerous tissues	1.26	*P* > 0.0125
Carcinous tissues and non-carcinous tissues	0.07	*P* > 0.0125
adjacent cancerous tissues and non-carcinous tissues	0.45	*P* > 0.0125

No significant differences were showed among three groups or the combination of any two groups via χ2 tests. Thus, we could draw a conclusion that the *SSTR-3* mRNA expression among carcinous tissue, adjacent tissue of cancer, and non-carcinous tissue were similar (Table 4).

We concluded that the expression of *SSTR-2* was significantly associated with survival rates (Table 1). The survival rate of patients with a positive expression was higher than those with a negative expression (*P* < 0.05*). The expression of *SSTR-2* mRNA and *SSTR-3* mRNA were relevant with gender. The female positive rate was significantly higher than males (*P* < 0.05**). These three kinds of mRNA’s expressions had no obvious relationships with age, TNM stage, and cancer differentiation (*P* > 0.05).

**Figure 1:**
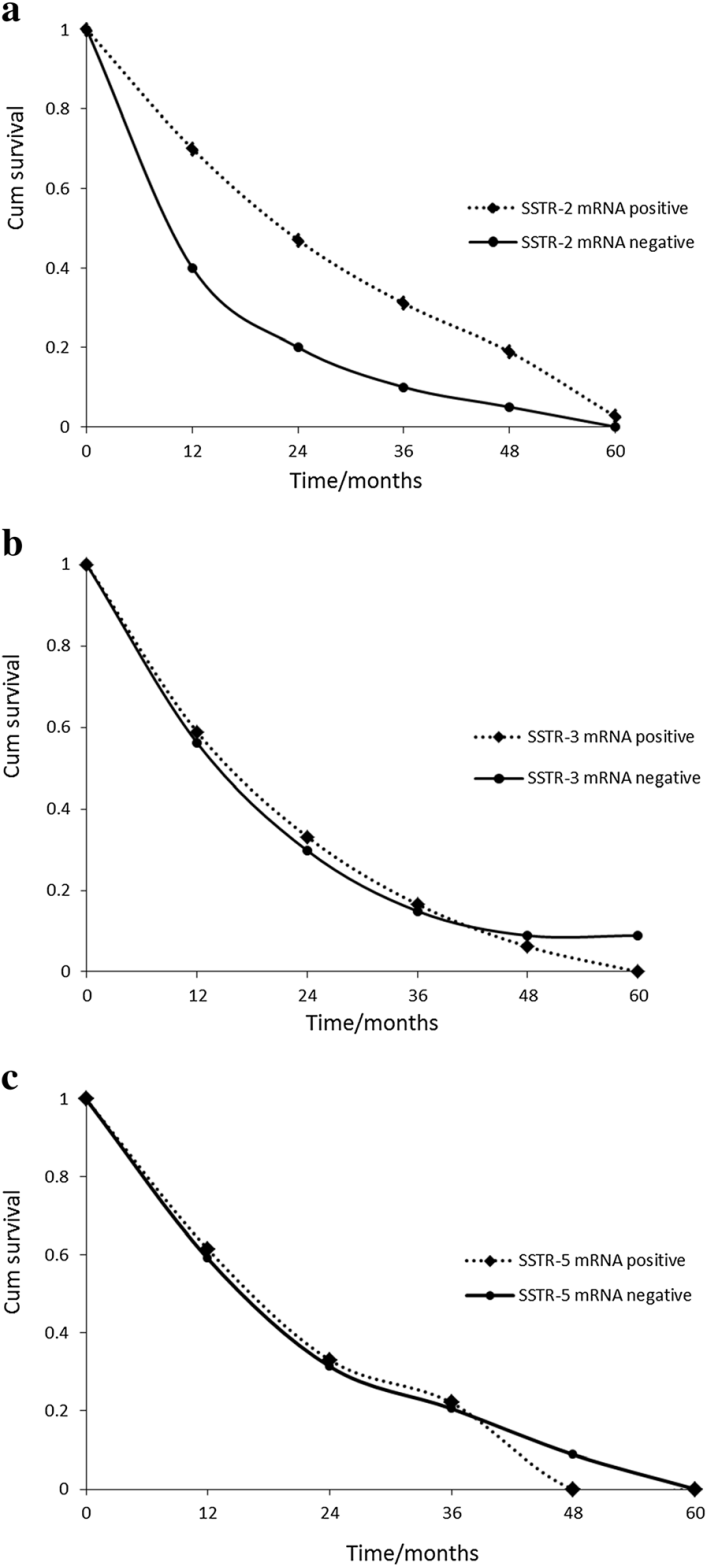
**Overall survival according to** ***SSTR-2,SSTR-3,SSTR-5*** **mRNA expression in pancreatic cancer patients. (a)** Overall survival according to *SSTR-2* mRNA expression in pancreatic cancer patients. **(b)** Overall survival according to *SSTR-3* mRNA expression in pancreatic cancer patients. **(c)** Overall survival according to *SSTR-5* mRNA expression in pancreatic cancer patients.

Survival curves are shown in Figure 1a-c. Patients with positive *SSTR-2* mRNA expression in carcinous tissue had a significantly better overall survival rate than those with negative expression (*P* < 0.05, the log-rank test, χ2 = 4.40). The difference of a patients’ survival rate between positive and negative *SSTR-3* expression in carcinous tissue had no statistical significance (*P* > 0.05, the log-rank test, χ2 = 0.003), and the difference of a patients’ survival rate between positive and negative *SSTR-5* expression in carcinous tissue also had no statistical significance (*P* > 0.05, the log-rank test, χ2 = 0.391).

## Discussion

Somatostatin can inhibit growth of pancreatic cancer cells even in CPT-insensitive human pancreatic carcinoid BON cells [6]. The inhibition mechanism involves the combined interaction of somatostatin and its analogs with *SSTR-2* in tumor tissues, mainly through binding to tumor tissue receptors [7], which directly inhibits tumor cell proliferation. This occurs through the process of either directly inhibiting division and proliferation of tumor cells or inhibiting the activity of various growth factors, such as vascular endothelial growth factor (*VEGF*), insulin-like growth factor (IGF) and marked stimulation of the reticuloendothelial system [8,9]. Moreover, it counteracts tumorigenesis and tissue proliferation. In addition, it was found that somatostatin and its analogs could arrest the pancreatic cancer cell cycle at S phase and thus induce apoptosis [10]. In cloned somatostatin receptor subtypes, synthetic long-acting somatostatin analogs 6 or 8, such as octreotide and vapreotide have high affinity to *SSTR-2* and *SSTR-5* receptor subtypes, low affinity to *SSTR-3*, and no affinity to other types of receptor subtypes [11]. Therefore, before implementing therapy by long-acting somatostatin analogs such as octreotide in pancreatic cancer patients, to make sure that tumor tissue have high affinity somatostatin receptors, it is important to improve efficacy and reduce unnecessary costs. There is genre and organ specificity in the expression of somatostatin receptors; for example, the normal pancreas mainly expresses *SSTR-2* subtype receptors, while only *SSTR-3* genes are expressed in rat pancreatic tissue; in the outer periphery of the body organization, only *SSTR-5* subtype receptors are found in the hypothalamus and pituitary tissue [12]. Therefore, in relation to drug receptor binding affinity and organ-specific aspects, we studied *SSTR-2*, *SSTR-3*, and *SSTR-5* expression in the pancreatic cancer.

There are few reports about research on somatostatin receptor gene expression in pancreatic tumor tissue. Somatostatin receptor expression in pancreatic tissue is detected by using isotopically labeled somatostatin [13]. However, because natural or synthetic somatostatin can bind to various somatostatin receptors, we cannot determine which kind of somatostatin subtype receptor binds to isotopically labeled somatostatin. RT-PCR assay has good specificity and high sensitivity for detection of *SSTR-2* gene expression in tumor specimens.

From the results of this study, there were 88 cases with positive expression of *SSTR-2* mRNA out of 108 pancreatic cancer cases; thus, the rate was 81.5%. This shows that the majority of pancreatic tumor tissues have *SSTR-2* receptor subtype expression. Because the current study shows that the ability of somatostatin to inhibit tumor cell growth is mediated by *SSTR-2* [14,15], it is very important for patients suffering from pancreatic cancer to receive somatostatin treatment. In experimental pancreatic tumor, the growth of both functioning pancreatic tumors and nonfunctioning tumors with *SSTR-2* gene expression is inhibited by somatostatin, while there is no inhibitory effect on non-*SSTR-2* gene expression cells. Carcinoid patients had a similar situation [16]. Researchers believe that the expression of *SSTR-2* in tumor tissue is the premise for patients to accept somatostatin therapy. In addition, *SSTR-3* mRNA expression was higher in pancreatic tumor tissue than the adjacent tissue of cancer, and the rate of positive expression decreases with increasing degree of differentiation trend. Significance of pancreatic tissue *SSTR-3* mRNA expression is still unclear. Compared with *SSTR-2*, *SSTR-3* has low affinity when compared to six or eight peptide long-acting somatostatin analogs, so further study is needed to determine its role in the treatment of tumors.

There is less difference in the rate of *SSTR-5* mRNA expression in pancreatic tumor tissue and tumor adjacent tissue, both were low, which is consistent with previous findings [17]. Synthetic six or eight long-acting somatostatin analogs like octreotide and vapreotide have high affinity to the *SSTR-5* receptor subtype [10]; its specific mechanism in pancreatic cancer remains to be further explored.

## Conclusions

In summary, our findings indicated that the majority of pancreatic cancers have more than one somatostatin receptor subtype gene. According to recent studies, we obtained that somatostatin and its analogs have obvious anti-proliferative effects on various solid tumors which are mediated through somatostatin receptors. We propose that the *SSTR-2* could play an important role in clinical implications for patients with pancreatic cancer undergoing somatostatin or its analog therapy.

## Abbreviations

SSTR: somatostatin receptor
RT-PCR: reverse transcriptase polymerase chain reaction
PNETs: pancreatic neuroendocrine tumors
VEGF: vascular endothelial growth factor
IGF: insulin-like growth factor
